# Cardiovascular diseases and intersectionality: beyond traditional risk factors

**DOI:** 10.1590/1677-5449.202500522

**Published:** 2025-06-09

**Authors:** Vanessa Prado dos Santos

**Affiliations:** 1 Universidade Federal da Bahia – UFBA, Salvador, BA, Brasil.

Non-transmissible chronic diseases (NTCDs) were responsible for more than 70% of all deaths worldwide in 2017 and cardiovascular diseases (CVDs) constitute the number one cause of mortality.^1^ In turn, ischemic heart disease (IHD) was the main cause of death from CVD and, in conjunction with stroke, accounted for 84.9% of deaths from CVD worldwide.^1^ Ischemic heart disease, stroke, and peripheral arterial disease (PAD) are the three major diagnoses related to CVDs and atherosclerosis is the most common etiology.^2^ Advanced age, diabetes mellitus (DM), hypertension (high blood pressure), and smoking are some of the genetic and environmental risk factors associated with CVDs.^2,3^ With regard to the behavior of theserisk factors,from 2010to 2019,there was a reductionin exposure to smoking and a greater than 1% per annum increase in exposure to hyperglycemia.^4^ Globally, the risk ofexposure to hyperglycemiaincreased significantly, by 1.32% peryear, whereas smokingreduced by 1.2%.^4^ Exposure to hypertension also increased, although to a lesser extent, withan annual increase of 0.51%.^4^In Brazil, the prevalence of hypertension in the adult populationis 32.3%, reaching 71.7% among people over the age of 70 years.^5^ Diabetes mellitus constitutes a serious public health problem all over the world, with prevalence that is growing year-on-year in many different countries.^6-8^ In 2018, it was estimated that more than 34 million people in the United States had DM, equating to 13% of the adult population and accounting for 26.6% of those aged 65 years or over.^7^ In Brazil, the estimated prevalence is 9.2%, at 8.1% of men and 10.2% of women.^8^

While genetic and environmental factors are relevant, studies indicate that they alone do not affect the risk of sickness. In the social sciences, the term “intersectionality” was proposed by Crenshaw (1989) to describe the understanding of the complexity of the interactions between different economic and social characteristics, emphasizing the importance of analysis of ethno-racial and social factors.^9,10^ Varied methodologies have been developed to study intersectionality in the social and health sciences, revealing the complexity of interactions between different life conditions among social strata, genders, and ethnic groups.^9-13^ In Brazil, one multicenter study found an increased risk of CVD-related death among people with black or brown skin color/race.^11^ An intersectional analysis combining skin color and sex detected a 52% increase in risk of mortality among white men, a 96% increase among brown-skinned men, and a 118% increase among black men.^11^ This finding reinforces the need to conduct analyses that consider interactions among several factors, such as ethno-racial, gender, economic, and social variables.

Studying the interactions between gender, ethno-racial, and socioeconomic characteristics through the lens of intersectionality can yield more in-depth analyses of the risk of CVDs, including PAD, and facilitate understanding of many aspects of prevention and treatment. A study conducted in Sweden showed that there was a greater risk of ischemic cardiac disease (ICD) among men. However, when income was included in the analysis, it was observed that risk was higher among people with low income, with 1.69 times higher risk among men and 2.19 times higher risk among women.^14^ Moreover, men and women with low income who lived alone and had lived in Sweden for 10 years or less had an even higher risk of ICD.^14^ In Spain, a study of cardiovascular mortality analyzed sex, age, and educational level, finding an inverse association between cardiovascular mortality and educational level, especially among women, 8.9% of whom had not completed primary education, compared with 6.2% of men.^15^ The difference in cardiovascular mortality between those with the lowest and highest educational levels was 88% for women and 44% for men.^15^

The literature also reveals associations between certain social and ethnic groups and greater vulnerability to vascular diseases and amputations.^16,17^ In 2023, a retrospective study of critical lower limb ischemia found an elevated risk of major amputations among Black/African-American and Latino/Hispanic patients, irrespective of the severity of clinical status at admission, as assessed using the WIfI Classification.^18^ In Australia, patients living in more economically vulnerable circumstances exhibited a higher incidence of advanced stage PAD, with complications such as ulcers and gangrene, in addition to higher rates of amputation.^19^ With regard to the impact of gender, while studies have not confirmed worse revascularization outcomes among women,^20,21^ some studies have identified higher rates of complications related to vascular access.^20^ Others have identified a higher risk of graft failure in Black women who undergone vascularization.^22^ The literature has suggested new interactions between a range of different risk factors, mortality, and CVDs in different parts of the body. A population study with elderly Japanese people (≥ 65 years) revealed that those whose routines involved greater social isolation exhibited higher all-causes mortality (odds ratio 1.20; 95% confidence interval [95%CI] 1.09-1.32) and mortality due to malignant neoplasms (odds ratio 1.14; 95%CI 1.01-1.28). However, higher CVD mortality was not confirmed (odds ratio 1.22; 95%CI 0.98-1.52) in this Japanese study.^23^

The 2024 European Society for Vascular Surgery (ESVS) guidelines discuss socioeconomic, ethnic, and gender aspects in relation to PAD.^24^ According to this publication, studies of gender and PAD indicate later onset of symptoms among women, but report conflicting results, and there is a need to include more women in future studies.^21^ With regard to socioeconomic and ethnic aspects, the literature reports greater PAD prevalence in lower income countries and also among groups with lower income and educational level and also suggests that outcomes are worse among people of African-American ethnicity.^24^ Brazil has an area of 8,510,417.771 km^2^, encompassing many different geographic and environmental conditions, which are compounded by marked economic, social, ethnic, and cultural differences.^25^ Ethno-racial characteristics, educational level, and income, in combination with genetic and environmental risk factors, act in a complex manner on the risk of sickness due to many different etiologies, including CVDs. Daily, while practicing our specialty, we perceive the complex interaction between the different risk factors of the peripheral vascular diseases and we have the opportunity to reflect on the influence of living conditions on the health of Brazilian population.
