# Supplementary material for: Cardiovascular diseases and intersectionality: beyond traditional risk factors
Source: J Vasc Bras. 2025 Jun 9;24:e20250052. doi: 10.1590/1677-5449.202500522 (PMC12186682; doi:10.1590/1677-5449.202500522)
Supplement: Translation

# Translation: Doenças cardiovasculares e interseccionalidade: além dos fatores de risco tradicionais

**DOI:** 10.1590/1677-5449.202500521

**Authors:** Vanessa Prado dos Santos

**Affiliations:** 1 Universidade Federal da Bahia – UFBA, Salvador, BA, Brasil.

As doenças crônicas não transmissíveis (DCNTs) foram responsáveis por mais de 70% dos óbitos mundiais em 2017, com as doenças cardiovasculares (DCVs) figurando como a principal causa de mortalidade^1^ . A doença cardíaca isquêmica (DCI) foi a principal causa de morte por DCV e, em conjunto com o acidente vascular encefálico (AVE), representou 84,9% dos óbitos por DCV no mundo^1^ . A DCI, o AVE e a doença arterial periférica (DAP) são os três principais diagnósticos relacionados às DCVs, que têm na aterosclerose sua etiologia mais comum^2^ . Idade avançada, diabetes mellitus (DM), hipertensão arterial sistêmica (HAS) e tabagismo são alguns dos fatores de risco genéticos e ambientais associados às DCVs^2,3^ . Quanto ao comportamento desses fatores de risco, entre 2010 e 2019, houve uma redução na exposição ao tabagismo e um aumento superior a 1% ao ano na exposição à hiperglicemia^4^ . Globalmente, o risco de exposição à hiperglicemia apresentou um aumento significativo de 1,32% por ano, enquanto o tabagismo declinou em 1,2%^4^ . A exposição à HAS também aumentou, porém em menor escala, com um crescimento anual de 0,51%^4^ . No Brasil, a prevalência de HAS na população adulta é de 32,3%, atingindo 71,7% entre indivíduos com mais de 70 anos^5^ . O DM representa um grave problema de saúde pública no mundo todo, com prevalência em crescimento ao longo dos anos em diversos países^6-8^ . Em 2018, nos EUA, estimava-se que mais de 34 milhões de pessoas tinham DM, correspondendo a 13% da população adulta, chegando a 26,6% entre aqueles com 65 anos ou mais^7^ . No Brasil, a prevalência estimada de DM é de 9,2%, sendo de 8,1% entre os homens e 10,2% entre as mulheres^8^ .

Embora relevantes, estudos indicam que fatores genéticos e ambientais não atuam de maneira isolada no risco de adoecimento. Nas ciências sociais, o termo “interseccionalidade”, proposto por Crenshaw (1989), refere-se à compreensão da complexidade da interação entre diferentes aspectos econômicos e sociais, enfatizando a importância da análise dos fatores de gênero e étnico-raciais^9,10^ . Diversas metodologias têm sido desenvolvidas para o estudo das interseccionalidades nas ciências sociais e da saúde, evidenciando a complexidade da interação entre as diferentes condições de vida entre estratos sociais, gêneros e etnias^9-13^ . No Brasil, um estudo multicêntrico identificou maior risco de morte associada às DCVs entre pessoas de raça/cor preta ou parda e, realizando uma análise interseccional entre cor da pele e gênero, observou um aumento de 52% no risco de mortalidade entre homens brancos, 96% entre homens pardos e 118% entre homens pretos^11^ . Esse achado reforça a necessidade de análises que considerem a interação entre vários fatores, entre eles os étnico-raciais, de gênero, econômicos e sociais.

Estudar a interação das características de gênero, étnico-raciais e socioeconômicas sob a ótica da interseccionalidade pode aprofundar a análise sobre o risco das DCVs, incluindo a DAP, e auxiliar na compreensão de diferentes aspectos sobre a sua prevenção e tratamento. Na Suécia, um estudo mostrou que o risco de doença cardíaca isquêmica (DCI) foi maior entre os homens^14^ . No entanto, ao associar a esta análise o fator renda, observou-se que o risco era mais elevado em pessoas de baixa renda, sendo 1,69 vezes maior para os homens e 2,19 vezes maior para as mulheres^14^ . Além disso, homens e mulheres de baixa renda que moravam sozinhos e viviam no país há 10 anos ou menos apresentaram um risco ainda maior de DCI^14^ . Na Espanha, um estudo a respeito da mortalidade cardiovascular analisou sexo, idade e nível educacional, encontrando uma associação inversa entre mortalidade cardiovascular e escolaridade, especialmente entre as mulheres^15^ . Entre elas, 8,9% não tinham completado a educação primária, em comparação com 6,2% dos homens. A variação entre os índices de mortalidade cardiovascular entre as pessoas com os menores e os maiores níveis educacionais foi de 88% para mulheres e 44% para homens^15^ .

A literatura também aponta uma associação entre determinados grupos sociais e étnicos e uma maior vulnerabilidade às doenças vasculares e amputações^16,17^ . Em 2023, um estudo retrospectivo sobre isquemia crítica de membro inferior identificou um risco elevado de amputações maiores entre pacientes negros/afro-americanos e latinos/hispânicos, independentemente da gravidade do quadro clínico à admissão, avaliada pela Classificação WIfI^18^ . Na Austrália, pacientes em condições socioeconômicas mais vulneráveis apresentaram maior incidência de DAP em estágio avançado, com complicações como úlceras e gangrena, além de taxas mais altas de amputação^19^ . Quanto ao impacto do gênero, embora os estudos não confirmem piores desfechos das revascularizações para as mulheres^20,21^ , alguns apontam que entre elas há uma maior taxa de complicações relacionadas ao acesso vascular nas intervenções^20^ . Outros autores identificaram um risco mais alto de falência do enxerto em mulheres negras submetidas às revascularizações do membro inferior^22^ . A literatura tem sugerido novas interações entre diversos fatores de risco, mortalidade e DCVs em seus diferentes territórios. Um estudo populacional realizado com idosos japoneses (≥ 65 anos) revelou que aqueles com rotinas de maior isolamento social apresentaram maior mortalidade por todas as causas (*odds ratio* 1,20; intervalo de confiança de 95% [IC95%] 1,09-1,32) e também por neoplasias malignas (*odds ratio* 1,14; IC95% 1,01-1,28).^23^ No entanto, nesse estudo a maior mortalidade por DCVs não foi comprovada (*odds ratio* 1,22; IC95% 0,98-1,52)^23^ . 

Na diretriz da European Society for Vascular Surgery (ESVS) de 2024, aspectos socioeconômicos, étnicos e de gênero são mencionados em relação à DAP^24^ . De acordo com a publicação, os estudos sobre gênero e DAP indicam um início mais tardio dos sintomas entre as mulheres, mas apresentam resultados conflitantes, sendo necessária uma maior inclusão de mulheres em pesquisas futuras^21^ . Quanto aos aspectos socioeconômicos e étnicos, a literatura aponta uma maior prevalência de DAP em países de menor renda, bem como entre grupos de menor renda e escolaridade, sugerindo também piores desfechos no tratamento para a etnia afro-americana^24^ . O Brasil é um país de 8.510.417,771 km^2^, com aspectos geográficos e ambientais distintos, que se somam a marcadas diferenças econômicas, sociais, étnicas e culturais^25^ . Características étnico-raciais, escolaridade e renda se combinam a fatores de risco genéticos e ambientais, atuando de maneira complexa no risco de adoecimento por diversas etiologias, incluindo as DCVs. Diariamente, ao atuar na especialidade, percebemos a interação complexa dos diversos fatores de risco associados às doenças vasculares periféricas e temos a oportunidade de refletir sobre a influência das condições de vida na saúde da população brasileira.
